# Perspective: Current Scientific Evidence and Research Strategies in the Role of Almonds in Cardiometabolic Health

**DOI:** 10.1016/j.cdnut.2024.104516

**Published:** 2024-11-28

**Authors:** Paula R Trumbo, Jamy Ard, France Bellisle, Adam Drewnowski, Jack A Gilbert, Ronald Kleinman, Anoop Misra, John Sievenpiper, Maha Tahiri, Karol E Watson, James Hill

**Affiliations:** 1 Paula R. Trumbo Consulting, Mount Pleasant, SC, United States; 2 School of Health Sciences, Liberty University, Lynchburg, VA, United States; 3 Division of Public Health Sciences, Department of Epidemiology and Prevention, School of Medicine, Wake Forest University, Wake Forest, NC, United States; 4 Nutritional Epidemiology, University of Paris, Bobigny, France; 5 Center for Public Health Nutrition, University of Washington, Seattle, WA, United States; 6 Department of Pediatrics, University of California San Diego, San Diego, CA, United States; 7 Scripps Institution of Oceanography, University of California San Diego, San Diego, CA, United States; 8 Department of Pediatrics, Massachusetts General Hospital, Harvard Medical School, Boston, MA, United States; 9 Fortis C-DOC Center for Diabetes, Obesity, Metabolic Diseases, and Endocrinology, New Delhi, India; 10 National Diabetes Obesity and Cholesterol Foundation, and Diabetes Foundation, India; 11 Department of Nutritional Sciences and Medicine, Temerty Faculty of Medicine, University of Toronto, Toronto, ON, Canada; 12 Division of Endocrinology and Metabolism, Department of Medicine, St Michael’s Hospital, Toronto, ON, Canada; 13 Li Ka Shing Knowledge Institute, St. Michael’s Hospital, Toronto, ON, Canada; 14 Nutrition Sustainability Strategies, St Petersburg, FL, United States; 15 Division of Cardiology, David Geffen School of Medicine, University of California, Los Angeles, CA, United States; 16 Department of Nutrition Sciences, Nutrition Obesity Research Center, University of Alabama at Birmingham, Birmingham, AL, United States

**Keywords:** almonds, blood pressure, LDL cholesterol, glycemic response, microbiome, metabolic syndrome, cardiometabolic health

## Abstract

Almonds are consumed by individuals around the world. Because almonds are rich in protein, unsaturated fatty acids, and fiber, a significant amount of research has been conducted on their role in affecting various cardiometabolic endpoints (body weight, blood pressure, blood cholesterol levels, and glycemic response). The most current meta-analyses on almond consumption and various health-related endpoints suggest that almond consumption does not result in weight gain and results in small reductions in LDL cholesterol and diastolic blood pressure, as well as improved glycemic responses in certain populations (i.e. Asian Indians). A number of research gaps on almond consumption and cardiometabolic health were identified that should be addressed to further understand their role in the various cardiometabolic endpoints, including the mechanisms of action interactions with the microbiome with regular consumption and their role as part of a healthy dietary pattern for both individuals and the general population.

## Introduction

The almond is a tree nut and its whole form (e.g. natural, roasted) is commonly consumed as a snack or integrated into meals. In addition, there are various other forms of almonds that are consumed as a routine part of a daily diet. Almond flour is finely ground almonds that—is sold in its natural or defatted form and is used as an alternative or complement to other flours. Almond “milk” is the liquid from almonds that have been ground in water and drained and is a popular dairy-free beverage. Almond butter is usually composed of almonds that have been blended without any non–almond-derived oil and is typically used as a spread, dip, or component of baked goods. Almond butter has a similar consistency to peanut butter and may serve as an alternative for those with a peanut allergy. Whole blanched almonds, along with other ingredients, are used to make almond paste for baking.

It has been estimated that the average annual consumption of whole almonds in the United States is 2.3 lb (1.04 kg) per person, with roasted whole almonds being most commonly consumed. Similarly, European countries, including France, Italy, and Germany, consume an estimated 1.6 lb (0.73 kg) to 2.3 lb (1.04 kg) annually per person, the most common form being natural. In Asian countries such as India, South Korea, China, and Japan, the estimated annual almond consumption is lower at 0.2 lb (0.09 kg) to 1.3 lb (0.59 kg) per person.

A 28 g (1/4 cup) serving size of raw, whole almonds provides ∼165 calories, 6 g of protein, 14 g of fat (67% MUFA, 11% PUFA, and 8% saturated), and 3 g of dietary fiber [1]. Almonds are part of the nuts, seeds and soy products subgroup to the protein food group of the USDA US Style eating patterns. The 2020–2025 Dietary Guidelines for Americans identified nuts as protein food that makes up a healthy eating pattern [2].

Whereas most metrics that attempt to capture the nutrient density of foods are based on the presence of nutrients of concern (sodium, saturated fats, and cholesterol), some nutrient profiling methods also include positive nutrients of public health significance such as protein, dietary fiber, calcium, and potassium [3]. Specifically, the Nutrients Rich Food Index (NRF 9.3) calculated per 100 g gives especially high scores to almonds and tree nuts compared with other foods in the USDA protein foods group. Almonds score high because of the high content of dietary fiber, protein, vitamins, and minerals and low content of saturated fatty acids, added sugars, and sodium. Calculated per 100 g, almonds had the highest mean NRF score compared with all other foods in the protein food group [3].

Much research has been conducted to understand how almonds influence human health and disease, including cardiovascular disease (CVD), diabetes risk, and body weight/obesity. There has been much interest in investigating the effect of almond consumption on these endpoints because of some of the components found in almonds. The Food and Drug Administration (FDA) issued a qualified health claim on nuts, including almond consumption and risk of coronary artery disease [4]. Almonds contain *β*-sitosterol, a phytosterol for which there is well-established evidence that it can lower LDL cholesterol levels [5]. As such, the FDA issued a health claim regulation for phytosterols and risk of CVD based on LDL cholesterol data. Furthermore, almonds contain by weight 67% monounsaturated fats, 11% polyunsaturated fats, and 11% dietary fiber. There is strong evidence that polyunsaturated fats reduce the risk of heart disease when replacing saturated fats [6], as well as some evidence that monounsaturated fats from various sources replace saturated fats in the diet [7]. Possibly due to the high fat (54%) and protein (20%) content of almonds, their consumption has been shown to enhance the feeling of fullness and satiety and, therefore, may lead to energy compensation and decrease overall daily calorie intake from other foods [8].

The nutritional composition of almonds has led to their inclusion in numerous randomized clinical trials to evaluate their role in various health and disease markers. Almonds have also been studied extensively in observational studies. However, unlike other nuts or nuts as a whole, almonds are not listed individually in most validated food frequency questionnaires (FFQ). That omission limits the utility of large-scale observational studies that use FFQ data to evaluate the association between almond consumption and health outcomes.

Using primarily published meta-analyses that specifically evaluated clinical trials on almonds, this article presents the perspectives of an Expert Panel of scientists convened by the Almond Board to evaluate the current state of the science regarding almond consumption and its effects on CVD, diabetes risk, and weight gain/obesity. In addition, the Expert Panel was charged with identifying priorities for future research in this area.

## Anthropometric Endpoints

Two meta-analyses considered studies of the impact of almond consumption on body weight and body composition [9,10] and found similar results. Studies included in these meta-analyses used almond doses of 10–100 g/d for periods ranging from 3 wk to 18 mo. Studies included those where almonds replaced other calories in the diet, as well as those where almonds were added to the diet. Study participants included a wide range of body mass indices. There was an impressive consistency in the lack of weight gain seen across all studies. In fact, there was some evidence that almond consumption of ≥50 g/d may have led to small reductions in body weight in some study participants.

Energy density has been historically measured using the Atwater factors [11]; however, for some foods, such as almonds, this method may overestimate the energy density because it does not account for the lower digestibility of nuts. Unlike other foods, the macronutrients in nuts, especially fats, are less available for digestion because of intact cell walls that encapsulate nutrients, making them less accessible to digestive enzymes. A rigorous study designed to test this overestimation [12] has shown that a significant portion of the fats in whole nuts is excreted rather than absorbed, resulting in a lower actual metabolizable energy (ME) than the values predicted by the Atwater factors. Consequently, the empirically available energy density of almonds (4.6 kcal/g) is substantially lower than the energy density calculated using the Atwater factors (6.1 kcal/g). That overestimation has consequences for nutrient profiling systems that are often based on the nutrients to calorie ratio. Nutrient density of almonds will be higher when calculated using revised energy content values.

The lack of weight gain that might be expected in studies where almonds were added to the diet suggests that, besides the lower ME, almond consumption must stimulate compensation for additional calories. This could potentially be a result of reduced energy intake from other sources, incomplete absorption of fat, microbiome effects, and/or increases in energy expenditure. Understanding how this compensation occurs is a high priority for future research.

## Risk of Cardiovascular Disease

Numerous studies and meta-analyses have addressed the effect of almond consumption on LDL cholesterol. The original trials have generally been relatively small, using both substitution and addition of almonds to the diets and involving study participants with a wide range of body mass indices. As with the studies focused on body weight, these studies used almond doses of 20 to 113 g/d, and the duration of the almond consumption period ranged from 4 weeks to 18 months. In some cases, effects of almonds on LDL cholesterol were significant, and in others not. While there was great variability in the findings among the studies, the studies trended toward a significant pooled reduction in LDL cholesterol (−0.124; 95% CI: −0.196, −0.051), reflecting a 5.1 mg/dL reduction. Based on the review of the published data, including 3 meta-analyses [[13], [14], [15]], almond consumption results in a reduction of LDL cholesterol by ∼5 mg/dL for the general population, including those at risk of chronic diseases.

Another meta-analysis of 5 studies conducted in individuals with type 2 diabetes did not show a significant pooled effect on LDL cholesterol [16]. This may have been the result of the limited number of studies, which ranged in sample size from 20 to 33 subjects. When subgroup analysis was conducted, however, there was a significant reduction in LDL cholesterol (−12.07; 95% confidence interval [CI]: −21.78, −2.35) for those who consumed >50 g/d almonds.

## Blood Pressure

Similar to LDL cholesterol, numerous intervention studies have been conducted on the effect of almond consumption on blood pressure. These studies are generally small, with varying doses of almonds consumed for varying amounts of time. Some studies used almond substitution, and others added almonds to the diet. Despite the variability in studies, 3 meta-analyses [15,17,18] of almonds and blood pressure have been conducted. Two of these [15,17] found very small (1.3 mm, 0.17 mm), but significant decreases in diastolic blood pressure. All 3 meta-analyses found no effect of almond consumption on systolic blood pressure.

There was some indication of greater effects with a longer feeding period (10+ wks) and a higher dose of almonds (50+ g) among these studies. Thus, there is a great need for studies with sufficient sample size to evaluate the impact of almonds on blood pressure in different subpopulations, including older adults, those with higher baseline blood pressure, and those with obesity.

## Risk of Diabetes

Three meta-analyses were identified that included trials that analyzed the effect of almond consumption on risk biomarkers of type 2 diabetes [i.e. fasting blood glucose (FBG), Hemoglobin A1c (HbA1c], and insulin resistance using the HOMA method) [15,19,20]. None of these meta-analyses showed a significant effect of almond consumption on FBG. The number of studies included in each of these meta-analyses ranged from 6 to 23 and included healthy individuals and individuals with type 2 diabetes. Furthermore, a significant reduction in FBG was not observed for any of the subgroups analyzed [15,20].

The findings of the meta-analyses were mixed for HbA1c, with one observing a significant reduction with almond consumption (mean difference; −0.52; 95% CI: −0.58, −0.46) (Oja) and 2 observing no effect [15,20]. Although Ojo et al. [19] included only 6 studies, these studies were conducted exclusively on individuals with type 2 diabetes, whereas the other 2 meta-analyses were conducted on a mix of healthy individuals and individuals with type 2 diabetes. The results of Ojo et al. [19] also must be viewed with caution, as there appears to have been an extraction error with SEs extracted as SDs from 2 of the included trials [21,22], resulting in an overweighting of these 2 trials, which together contribute >90% of the weight to the pooled estimate. Morvaridzadeh et al. [15] did not observe a significant reduction in HbA1c among a subgroup of studies on type 2 diabetes; however, this analysis included only 2 studies.

None of the meta-analyses observed a significant change in insulin resistance-HOMA among the study intervention groups. Subgroup analysis showed evidence of effect modification by age, weight status, health status, duration of follow-up, and risk of bias with no clear pattern on risk factors for type 2 diabetes among participants [20].

## Risk of Diabetes in Asian Indians

The above discussed meta-analyses represented many countries, including the United States, Canada, Australia, Iran, and Asian countries such as Taiwan and Korea. It is recognized that South Asians have an increased susceptibility to type 2 diabetes and metabolic dysfunction [23]. Insulin resistant Asian Indians have significantly higher plasma insulin levels and associated IR-HOMA compared to insulin resistant Caucasians [24]. Furthermore, postprandial blood glucose levels are significantly higher in Asian Indians compared with other geographic locations [25]. Although meta-analyses have not been conducted specifically on almond consumption and Asian Indians, several feeding studies have been conducted on this population [[26], [27], [28], [29], [30]]. Three studies evaluated the effect of almond consumption (20 to 60 g/da) on FBG and HcA1C in Asian Indians with prediabetes [[27], [28], [29]]. These 3 studies reported that, compared with a control, there was a significant reduction in FBG (5.5, 6.3, and 6.1 mg/dL) and HbA1c (−0.09, −0.4, −0.4). Such findings were not observed in healthy, overweight individuals [30]. In individuals with type 2 diabetes, the findings were mixed in that there was a significant reduction in HbA1c, but not for FBG [26].

## The Effect of Almond Consumption on the Composition and Metabolome of the Microbiome

The relative proportions of the bacterial species that compose the gut microbiome can be significantly influenced by diet. The food consumed 2 d prior to sampling and analysis of a fecal sample tends to have the biggest impact on microbiome community structure, and the same food can have different effects on the microbiome of different people, suggesting individualized responses [31]. The microbiome has also been shown to influence metabolic health; for example, microbiome-derived short-chain fatty acids (SCFAs), which are produced as a byproduct of microbial fiber fermentation, can regulate the activity and secretion of the incretin hormone glucagon-like peptide-1 (GLP1), which plays an important role in regulating insulin secretion, glucagon release, and gastric mobility, thereby impacting glycemic control and appetite regulation [32]. In addition, certain gut bacteria such as *Bacteroides Akkermansia muciniphila, Lactobacillus,* and *Bifidobacterium* are known to produce metabolites, such as SCFAs, indoles, and secondary bile acids that mimic the action of the incretin hormone gastric inhibitory polypeptide (GIP), or enhance its secretion, thereby improving postprandial insulin response and glycemic control [33]. GIP also plays a role in lipid metabolism by promoting lipid storage and adipogenesis, and therefore, microbial-induced changes in GIP activity can influence adiposity, potentially aiding in the reduction of body fat and the prevention of being overweight and even obesity.

Although the number of studies and the size of the human populations investigated are very limited, most studies point to the fact that almond consumption appears to modulate the structure of the gut microbiome, promoting growth of beneficial bacteria and increasing the production of SCFAs such as butyrate [34]. A randomized controlled trial comparing an almond-based low carbohydrate diet with a low-fat diet in 45 participants with type-2 diabetes for 3 mo, found that the almond-based diet increased GLP1 levels and reduced symptoms of depression, which was associated with an increase in the proportion of SCFA-producing bacteria (*Roseburia, Ruminococcus,* and *Eubacterium*) [35]. Another trial with 18 adults reported that chopped or whole almonds increased the proportion of other predominantly beneficial bacterial genera such as *Roseburia, Lachnospira, Oscillospira*, and *Dialister* [36]. A randomized, controlled, parallel-arm, 8-wk intervention in 73 young adults found that almond snacking increased microbial diversity by 3%, as well as influenced the abundance of certain (e.g. lipid and carbohydrate) microbially derived metabolites in circulation [37]. Another clinical trial showed that whole almonds and ground almond (almond flour) consumption resulted in greater butyrate levels compared with a control, which suggests stimulation of microbial fermentation of almond fiber. However, there was no effect of either almond source on microbial biodiversity, suggesting that the stimulated microbial activity may not have been in the colon where the fecal material was being formed but possibly occurred in the small intestine [38]. A randomized controlled trial providing either 56 g of almonds, 10 g of almond skins, or a control (fructooligosaccharide) for 6 wk showed that both almond interventions increased both the proportion of *Bifidobacterium* and *Lactobacillus* and the activity of the microbial bile-acid hydrolases in stool, as well as decreased the proportion of the pathogen *Clostridium perfringens* [39].

The overall conclusions from these above studies are that almond consumption is important in stimulating specific bacterial growth and the production of SCFAs, indoles, and secondary bile acids, which may have an impact on adiposity and metabolic health.

## Advancing the Knowledge and Understanding of the Role of Almonds in Cardiometabolic Health

The Almond Board Cardiometabolic Roundtable identified various findings and knowledge gaps (Table 1) [40,41] related to the role of almond consumption in supporting healthy body weight and influencing CVD and diabetes risk. The beneficial findings are provided in Table 2. Although these findings were agreed upon by the roundtable experts, several areas were identified that require further research. In general, it was recognized that many of the studies reviewed were underpowered, and there was a large heterogeneity among studies. It was agreed among the Expert Panel that the basis for this variability between individuals and across studies should be evaluated to understand the reasons (e.g. mechanism-driven). Understanding the mechanisms would be useful for re-evaluating the existing publications.

**TABLE 1:** The beneficial effects of almond consumption.

Consuming almonds daily does not result in weight gain; tendency for higher almond intake to be associated with slight weight loss.
Consuming almonds daily results in a consistent, small, significant average reduction (5.1 mg/dL or ∼5%) based on mean population levels of LDL cholesterol concentrations in the United States.
Consuming almonds daily results in a small, significant average reduction (0,17–1.3 mm Hg) in diastolic blood pressure.
While almonds as a single food produce average reductions in LDL cholesterol and diastolic blood pressure that are clinically small for individuals, these reductions have significant public health benefits and can be meaningful for individuals when combined with the LDL cholesterol and blood pressure lowering effects of other foods as part of guidelines-based dietary patterns that target lower LDL cholesterol (e.g. Portfolio diet) [40] and blood pressure (e.g. DASH diet) [41].
Consuming almonds daily by Asian Indians with prediabetes can result in a significant reduction in fasting blood glucose and HbA1C.
Almond consumption results in an increase in the proportion of potentially beneficial bacteria in stool, as well as an increase in the abundance of microbial metabolites that are known to influence metabolic health.

**Table 2:** Research and Knowledge Gaps for Almond Consumption and cardiometabolic effects.

Basis for the large heterogeneity in findings for cardiometabolic endpoints
The amount of almonds that can be consumed without weight gain
Basis for the lack of weight gain with almond consumption
Effective dose for reduction in LDL cholesterol levels
Basis for differences in glycemic response to almonds in Asian Indians compared to other populations.
Development of food frequency questionnaire that specifically measures almond consumption and the conduct of prospective cohort studies that evaluate the association between almond consumption and clinical endpoints such as cardiovascular disease and type 2 diabetes.
Determination of the different types of dietary fibers in almonds and their role in cardiometabolic endpoints
Using the up-to-date methods to evaluate the independent effect of almonds on the microbiome
The role of almonds as part of recommended healthful diets and precision nutrition.

With respect to body weight, more research using large randomized controlled trials is needed to understand the dose of almonds that can be consumed without resulting in weight gain. Furthermore, information is needed to understand whether the lack of weight gain is a result of a change in satiety/appetite, whether compensation is such that total energy intake does not change, or whether there is an effect on metabolic expenditure. Large randomized controlled trials are needed to understand the doses at which almonds can reduce LDL cholesterol concentrations and diastolic blood pressure. These studies should be of sufficient duration to understand the long-term effects of almond consumption and population specifics (e.g. age, healthy, at risk, diseased) to determine those who are most likely to benefit from almond consumption.

In general, most studies have failed to find a beneficial effect of almond consumption on glycemic endpoints (i.e. FBS, HbA1c, HOMA-IR). However, studies on Asian Indians with prediabetes consistently showed that almond consumption reduced FBS and HbA1c [[25], [26], [27]]. The sample size of these studies was 60, 66, and 275 individuals, and the doses were 20 g or 56 g of almonds per day. Comparative studies are needed to understand the difference in glycemic response to almonds in Asian Indians compared with other populations.

Because validated FFQ estimate the intake of nuts, rather than specifically almonds, prospective observational studies are lacking for measuring the association between almond intake and clinical endpoints, such as CVD and type 2 diabetes. For this reason, validated FFQ that estimate intake of specific kinds of nuts would be valuable, as not all nuts are the same in terms of nutrient composition.

Different dietary fibers have different physiological effects, and therefore, it will be important to identify the different types of fibers present in almonds, t understand the potential effects on LDL cholesterol, as well as glycemic effects from almond consumption. Although studies do provide evidence suggesting that almond fiber stimulates microbial fermentation and the production of metabolites with known effects on host metabolism, the studies so far are in small population sizes and are primarily obtained using parallel randomized controlled trials. Cross-over studies on the effect of almond intake are needed to rule out differences in an individual’s microbiome profile, which may influence the power to observe changes in parallel studies. Cross-over studies would allow there to be an independent evaluation of the effects of almond consumption on the microbiome and its effects, such as short chain fatty acid production. Importantly, all microbiome studies have been limited to proportional investigations and limited observation density over time, which mean that the absolute abundance of specific bacterial species cannot be ascertained, and no understanding of the temporal dynamics of bacterial communities can be observed. New techniques have been developed that allow for the quantification of the cellular abundance of all bacterial features in metagenomic data [42], as well as the analysis of these features in every stool sample produced during a trial. Future studies adopting these strategies will allow for a more robust interpretation of the impact of almond consumption on microbial abundance dynamics, which will enable us to determine how the microbiome influences the variability in host response to almond consumption.

As mentioned before, among all nuts in that category, almonds are not specifically identified as a food to be encouraged for consumption as part of a dietary pattern [2]. Randomized clinical trials that provide diets that have significant evidence for health and wellness, such as the USDA dietary patterns [2] or the Dietary Approaches to Stop Hypertension eating plan [43], can be conducted to examine the influence of almonds on such diets for the general population. Furthermore, as precision nutrition [45] advances in tailoring dietary recommendations for specific health endpoints of individuals, almonds can be part of that investigation for endpoints such as body weight, blood LDL cholesterol, glycemic control, blood pressure, and the microbiome. It will be important to understand the nutritional differences between the different forms of almonds (e.g. raw almonds, almond milk, almond flour) and how they each affect the various health endpoints.

## Author contributions

The authors’ responsibilities were as follows – PRT designed, wrote, and edited the manuscript; JA, FB, AD, JG, JH, REK, MT, KEW, JS, and AM critically reviewed and edited the manuscript; all authors were responsible for the final content of the manuscript.

## Data availability

Data described in this manuscript, code book, or analytic code are not available and will not be made available because all information provided is based solely on the cited articles.

## Funding

This work was supported by the Almond Board of California.

This perspective article, in part, includes information from an Almond Cardiometabolic Roundtable held July 15 and 16, 2024, in Modesto, California, that included presentations and dialog among academia representing various areas of nutrition and health. PRT received funding to collect scientific information, participate in the roundtable, and prepare the manuscript. MT received funding to collect scientific information, onboard relevant experts, and Chair the roundtable. FB, JA, AD, JG, JH, RK, AM, JS, and KW received travel funding and honorarium to participate in the July 2024 meeting.

## Conflict of interest

During the past 5 years, PRT has served as a consultant to General Mills, PepsiCo, Johnson & Johnson, Nestle United States, Ocean Spray, GlaxoSmithKline, Tate & Lyle, Ingredion, Bioneutra, Lantmännen, Hayashibara, MycoTechnology, Quebec Maple Syrup Producers, Colgate Palmolive, Almond Board, Constellation Brands, Kappa Biosciences, Kodiak Cakes, Bay State Milling, Intertek, The Protein Brewery, 8Greens, GRAS Associates, ILSI North America, and Institute for the Advancement of Food and Nutrition Sciences. JOH serves on an advisory board for General Mills and is a member of the McCormick Science Institute. FB serves on scientific advisory panels for General Mills and the International Sweeteners Association. RK has served on an advisory board for General Mills, Ocean Spray and the Sesame Street Workshop, as a consultant to Arla Food Ingredients and as Board Chair of the Global Child Nutrition Foundation and Project Bread, Boston, MA, and a member of the Board of Directors of the Agricultural and Food Sciences Inst., the European Biomedical Research Institute, Salerno, the Genetic Literacy Project, the David Ortiz Children’s Fund and UNICEF Regional Board, New England. AD is the original developer of the Naturally Nutrient Rich (NNR) and the Nutrient Rich Food (NRF) nutrient profiling models and is or has been a member of scientific advisory panels for BEL, Lesaffre, Nestle, FrieslandCampina, National Pork Board, and Carbohydrate Quality Panel supported by Potatoes United States. AD has worked with Ajinomoto, Ayanabio, DSM-Firmenich, FoodMinds, KraftHeinz, Meiji, MS-Nutrition, Nutrition Impact LLC, Nutrition Institute, PepsiCo, Samsung, and Soremantec on quantitative ways to assess nutrient density of foods. JA reports research support from Nestle Healthcare Nutrition, Eli Lilly, Epitomee, UnitedHealth Group, Novo Nordisk, KVK Tech, and WW and serves as a consultant for Regeneron, Brightseed, Boehringer Ingelheim, Intuitive, Novo Nordisk, Eli Lilly, and WW. JLS received research support from the Canadian Foundation for Innovation, Ontario Research Fund, Province of Ontario Ministry of Research and Innovation and Science, Canadian Institutes of Health Research (CIHR), Diabetes Canada, American Society for Nutrition (ASN), National Honey Board (U.S. Department of Agriculture [USDA] honey “Checkoff” program), Institute for the Advancement of Food and Nutrition Sciences (IAFNS), Pulse Canada, Quaker Oats Center of Excellence, INC International Nut and Dried Fruit Council Foundation, The United Soybean Board (USDA soy “Checkoff” program), Protein Industries Canada (a Government of Canada Global Innovation Cluster), Almond Board of California, European Fruit Juice Association, The Tate and Lyle Nutritional Research Fund at the University of Toronto, The Glycemic Control and Cardiovascular Disease in Type 2 Diabetes Fund at the University of Toronto (a fund established by the Alberta Pulse Growers), The Plant Protein Fund at the University of Toronto (a fund which has received contributions from IFF among other donors), The Plant Milk Fund at the University of Toronto (a fund established by the Karuna Foundation through Vegan Grants), and The Nutrition Trialists Network Fund at the University of Toronto (a fund established by donations from the Calorie Control Council, Physicians Committee for Responsible Medicine, and Login5 Foundation). He has received food donations to support randomized controlled trials from the Almond Board of California, California Walnut Commission, Danone, Nutrartis, Soylent, and Dairy Farmers of Canada. He has received travel support, speaker fees, and/or honoraria from FoodMinds LLC, Nestle, Abbott, General Mills, Nutrition Communications, International Food Information Council (IFIC), Arab Beverage Association, International Sweeteners Association, Calorie Control Council, and Phynova. He has or has had ad hoc consulting arrangements with the Almond Board of California, Perkins Coie LLP, Tate & Lyle, Ingredion, and Brightseed. He is on the Clinical Practice Guidelines Expert Committees of Diabetes Canada, the European Association for the Study of Diabetes (EASD), the Canadian Cardiovascular Society (CCS), and the Obesity Canada/Canadian Association of Bariatric Physicians and Surgeons. He serves as an unpaid member of the Board of Trustees of IAFNS. He is a Director at Large of the Canadian Nutrition Society (CNS), founding member of the International Carbohydrate Quality Consortium (ICQC), Executive Board Member of the Diabetes and Nutrition Study Group (DNSG) of the EASD, and Director of the Toronto 3D Knowledge Synthesis and Clinical Trials foundation. His spouse is a former employee of Nestle Health Science and AB InBev. The rest of the authors have no conflicts of interest or financial relationships relevant to the article to disclose. MT served as a consultant for Once upon a Farm, the Almond Board of California, Mycotechnology, The Protein Brewery, Bactolife, Alt Collective, Alpha Site, Benson Hill, Sound Agriculture, Umiami, Melibio, Bolder, Brain, Noblegen, Calyxt, Center for Food Integrity, Circe Bioscience, Gingko Bioworks, Culinex, Clareo, Kraft Heinz, Food Strategy Associates, Novo Nordisk, Emerald Technology Ventures, LFE Capital, DIC Corporation, Perfeggt, Eli Life, Emerid, Formo, Gosh Foods, Jack and Bry, Narayan, Eggmented, Reality, More Foods, Sokowell, and John B. Sanfilippo & Son Inc.

## References

[1] U.S. Department of Agriculture (2024). https://fdc.nal.usda.gov/.

[2] U.S. Department of Agriculture and U.S. Department of Health and Human Services (2020). https://www.dietaryguidelines.gov/.

[3] Drewnowski A., Fulgoni V. (2008). Nutrition profiling of foods: creating a nutrient-rich food index. Nutr. Rev..

[4] US Food and Drug Administration (2003). https://wayback.archive-it.org/7993/20171114183724/https://www.fda.gov/Food/IngredientsPackagingLabeling/LabelingNutrition/ucm072926.htm.

[5] Code of Federal Regulations. 21 CFR 101.83 (2024). https://www.ecfr.gov/current/title-21/chapter-I/subchapter-B/part-101#101.83.

[6] US Food and Drug Administration (2004). https://www.fda.gov/food/food-labeling-nutrition/health-claim-notification-substitution-saturated-fat-diet-unsaturated-fatty-acids-and-reduced-risk.

[7] US Food and Drug Administration (2024). https://www.fda.gov/food/food-labeling-nutrition/qualified-health-claims-letters-enforcement-discretion.

[8] Paddon-Jones D., Westman E., Mattes R.D., Wolfe R.R., Astrup A., Westerterp-Plantenga M. (2008). Protein, weight management, and satiety. Am. J. Clin. Nutr..

[9] Chahibakhsh N., Rafieipour N., Rahimi H., Rajabi Nezhad S., Momeni S.A., Motamedi A. (2024). Almond supplementation on appetite measures, body weight, and body composition in adults: a systematic review and dose-response meta-analysis of 37 randomized controlled trials. Obes. Rev..

[10] Eslampour E., Moodi V., Asbaghi O., Ghaedi E., Shirinbakhshmasoleh M., Hadi A. (2020). The eﬀect of almond intake on anthropometric indices: a systematic review and meta-analysis. Food Funct.

[11] Food and Agriculture Organization (2003). https://www.fao.org/4/y5022e/y5022e04.htm#bm4.

[12] Novotny J., Gebauer S., Baer D. (2012). Discrepancy between the Atwater factor predicted and empirically measured energy values of almonds in human diets. Am. J. Clin. Nutr..

[13] Musa-Veloso K., Paulionis L., Poon T., Lee H.Y. (2016). The effects of almond consumption on fasting blood lipid levels: a systematic review and meta-analysis of randomised controlled trials. J. Nutr. Sci..

[14] Lee-Bravatti M.A., Wang J., Avendano E.E., King L., Johnson E.J., Raman G. (2019). Almond consumption and risk factors for cardiovascular disease: a systematic review and meta-analysis of randomized controlled trials. Adv. Nutr..

[15] Morvaridzadeh M., Qorbani M., Eshkiki Z.S., Estêvão M.D., Ganjaroudi N.M., Toupchian O. (2022). The effect of almond intake on cardiometabolic risk factors, inflammatory markers, and liver enzymes: a systematic review and meta-analysis. Phytother. Res..

[16] Wang P., Sheng Y., Samadi M. (2021). Effects of almond consumption on lipid profile in patients with type 2 diabetes: a systematic review and meta-analysis of randomised controlled trials. Arch. Physiol. Biochem..

[17] Eslampour E., Asbaghi O., Hadi A., Abedi S., Ghaedi E., Lazaridi A.V. (2020). The eﬀect of almond intake on blood pressure: a systematic review and meta-analysis of randomized controlled trials. Complement. Ther. Med..

[18] Mohammadifard M., Salehi-Abargouei A., Salas-Salvadó J., Guasch -Ferré M., Humphries K., Sarrafzadegan N. (2015). The effect of tree nut, peanut, and soy nut consumption on blood pressure: a systematic review and meta-analysis of randomized controlled clinical trials. Am. J. Clin. Nutr..

[19] Ojo O., Wang X.H., Ojo O.O., Adegboye A.R.A. (2021). The effects of almonds on gut microbiota, glycometabolism, and inflammatory markers in patients with type 2 diabetes: a systematic review and meta-analysis of randomised controlled trials. Nutrients.

[20] Asbaghi O., Moodi V., Neisi A., Shirinbakhshmasoleh M., Abedi S., Oskouie F.H. (2022). |The effect of almond intake on glycemic control: a systematic review and dose–response meta-analysis of randomized controlled trials. Phytother. Res..

[21] Hou Y.Y., Ojo O., Wang L.L., Wang Q., Jiang Q., Shao X.Y. (2018). A randomized controlled trial to compare the effect of peanuts and almonds on the cardio-metabolic and inflammatory parameters in patients with Type 2 diabetes mellitus. Nutrients.

[22] Sattar N., Gill J.M.R. (2015). Type 2 diabetes in migrant South Asians: mechanisms, mitigation, and management. Lancet Diabetes Endocrinol.

[23] Abate N., Chandalia M., Snell P.G., Grundy S.M. (2004). Adipose tissue metabolites and insulin resistance in nondiabetic Asian Indian men. J. Clin. Endocrinol. Metab..

[24] Milicevic Z., Raz I., Beattie S.D., Campaigne B.N., Sarwat S., Gromniak E. (2008). Natural history of cardiovascular disease in patients with diabetes: role of hyperglycemia. Diabetes Care.

[25] Gulati S., Misra A., Pandey R.M. (2017). Effect of almond supplementation on glycemia and cardiovascular risk factors in Asian Indians in North India with type 2 diabetes mellitus: a 24–week study. Metab. Syndr. Relat. Disord..

[26] Gulati S., Misra A., Tiwari R., Sharma M., Pandey R.M., Upadhyay A.D. (2023). Premeal almond load decreases postprandial glycaemia, adiposity and reversed prediabetes to normoglycemia: a randomized controlled trial. Clin. Nutr. ESPEN.

[27] S. Gulati, A. Misra, R. Tiwari, M. Sharma, R.M. Pandey, A.D. Upadhyay, et al., Beneficial effects of premeal almond load on glucose profile on oral glucose tolerance and continuous glucose monitoring: randomized crossover trials in Asian Indians with prediabetes, Eur. J. Clin. Nutr. 7(5):2(023) 7586–7595.. 10.1038/s41430-023-01263-1 PMC10169634 36732571

[28] J. Madan, S. Desai, P. Moitra, S. Salis, S. Agashe, R. Battalwar, et al. Effect of almond consumption on metabolic risk factors—glucose metabolism, hyperinsulinemia, selected markers of inflammation: a randomized controlled trial in adolescents and young adults, Front. Nutr. 8 (24) 668622. 10.3389/fnut.2021.668622 PMC8264510 34249987

[29] Gayathri R., Abirami K., Kalpana N., Manasa V.S., Sudha V., Shobana S. (2023). Effect of almond consumption on insulin sensitivity and serum lipids among Asian Indian adults with overweight and obesity– a randomized controlled trial. Front. Nutr..

[30] Johnson A.J., Vangay P., Al-Ghalith G.A., Hillmann B.M., Ward T.L., Shields-Cutler R.R. (2019). Daily sampling reveals personalized diet-microbiome associations in humans cell host. Microbe.

[31] Ecklu-Mensah G., Gilbert J.A., Devkota S. (2022). Dietary selection pressures and their impact on the gut microbiome. Cell. Mol. Gastroenterol. Hepatol.

[32] Srivastava S., Singh R. (2021). Oral administration of Lactobacillus casei and Bifidobacterium bifidum improves glucagon like peptide-1 (GLP-1 and glucose-dependent insulinotropic polypeptide (GIP) level in streptozotocin induced diabetic rats. Curr. Res. Food Sci. Nutr..

[33] Singar S., Kadyan S., Patoine C., Park G., Arjmandi B., Nagpal R. (2024). Effects of almond consumption on cardiovascular health and gut microbiome: a comprehensive review. Nutrients..

[34] Rem M., Zhang H., Qi J., Hu A., Jiang Q., Hou Y. (2020). An almond-based low carbohydrate diet improves depression and glycometabolism in patients with type 2 diabetes through modulating gut microbiota and GLP-1: a randomized controlled trial. Nutrients.

[35] Holscher H.D., Taylor A.M., Swanson K.S., Novotny J.A., Baer D.J. (2018). Almond consumption and processing affects the composition of the gastrointestinal microbiota of healthy adult men and women: a randomized controlled trial. Nutrients.

[36] Dhillon J., Newman J.W., Fiehn O., Ortiz R.M. (2023). Almond consumption for 8 weeks altered host and microbial metabolism in comparison to a control snack in young adults. J. Am. Nutr. Assoc..

[37] Creedon A.C., Dimidi E., Hung E.S., Rossi E.S., Probert C., Grassby T. (2022). The impact of almonds and almond processing on gastrointestinal physiology, luminal microbiology, and gastrointestinal symptoms: a randomized controlled trial and mastication study. Am. J. Clin. Nutr..

[38] Liu Z., Lin X., Huang G., Zhang W., Rao P., Ni L. (2014). Prebiotic effects of almonds and almond skins on intestinal microbiota in healthy adult humans. Anaerobe.

[39] Chiavaroli L., Nishi S.K., Khan T.A., Braunstein C.R., Glenn A.J., Mejia S.B. (2018). Portfolio dietary pattern and cardiovascular disease: a systematic review and meta-analysis of controlled trials. Prog. Cardiovasc. Dis..

[40] Chiavaroli L., Viguiliouk E., Nishi S.K., Blanco Mejia S., Rahelić D., Kahleová H. (2019). DASH dietary pattern and cardiometabolic outcomes: an umbrella review of systematic reviews and meta-analyses. Nutrients.

[41] Zaramela L.S., Tjuanta M., Moyne O., Neal M., Zengler K. (2022). synDNA-a synthetic DNA spike-in method for absolute quantification of shotgun metagenomic sequencing. mSystems.

[42] National Institutes of Health (2021). https://www.nhlbi.nih.gov/education/dash-eating-plan.

[43] De Toro-Martin J., Arsenault B.J., Despres J.-P., Vohl M.-C. (2017). Precision nutrition: a review of personalized nutrition approaches for the prevention and management of metabolic syndrome. Nutrients.

